# Family planning counseling and its associations with modern contraceptive use, initiation, and continuation in rural Uttar Pradesh, India

**DOI:** 10.1186/s12978-019-0844-0

**Published:** 2019-12-12

**Authors:** Nabamallika Dehingia, Anvita Dixit, Sarah Averbach, Vikas Choudhry, Arnab Dey, Dharmendra Chandurkar, Priya Nanda, Jay G. Silverman, Anita Raj

**Affiliations:** 1 Center on Gender Equity and Health, Division of Global Public Health, University of California, San Diego School of Medicine, 9500 Gilman Drive #0507, La Jolla, CA 92093-0507 USA; 2 Joint Doctoral Program, San Diego State University/University of California San Diego, San Diego, CA USA; 3 Department of Reproductive Medicine, University of California, San Diego, San Diego, CA USA; 4 Sambodhi Research and Communications Pvt. Ltd., Noida, India; 5 Bill and Melinda Gates Foundation, New Delhi, Delhi India

**Keywords:** Modern contraceptive use, Contraceptive continuation, FP counseling, FP quality, India

## Abstract

**Background:**

We examine the association between the quality of family planning (FP) counseling received in past 24 months, and current modern contraceptive use, initiation, and continuation, among a sample of women in rural Uttar Pradesh, India.

**Methods:**

This study included data from a longitudinal study with two rounds of representative household survey (2014 and 2016), with currently married women of age 15–49 years; the analysis excluded women who were already using a permanent method of contraceptive during the first round of survey and who reported discontinuation because they wanted to be pregnant (*N* = 1398). We measured quality of FP counseling using four items on whether women were informed of advantages and disadvantages of different methods, were told of method(s) that are appropriate for them, whether their questions were answered, and whether they perceived the counseling to be helpful. Positive responses to every item was categorized as higher quality counseling, vs lower quality counseling for positive response to less than four items. Outcome variables included modern contraceptive use during the second round of survey, and a variable categorizing women based on their contraceptive use behavior during the two rounds: continued-users, new-users, discontinued-users, and non-users.

**Results:**

Around 22% had received any FP counseling; only 4% received higher-quality counseling. Those who received lower-quality FP counseling had 2.42x the odds of reporting current use of any modern contraceptive method (95% CI: 1.56–3.76), and those who received higher quality FP counseling at 4.14x the odds of reporting modern contraceptive use (95% CI: 1.72–9.99), as compared to women reporting no FP counseling. Women receiving higher-quality counseling also had higher likelihood of continued use (ARRR 5.93; 95% CI: 1.97–17.83), as well as new use or initiation (ARRR: 4.2; 95% CI: 1.44–12.35) of modern contraceptives. Receipt of lower-quality counseling also showed statistically significant associations with continued and new use of modern contraceptives, but the effect sizes were smaller than those for higher-quality counseling.

**Conclusions:**

Findings suggest the value of FP counseling. With a patient-centered approach to counseling, continued use of modern contraceptives can be supported among married women of reproductive age. Unfortunately, FP counseling, particularly higher-quality FP counseling remains rare.

## Plain English summary

Providing high quality family planning (FP) counseling is important to support modern contraceptive use that meet couple’s needs. Improving the use of modern contraceptives in turn has potential to reduce unintended pregnancy and short inter-pregnancy intervals which lead to adverse health consequences for mothers and infants. We examined the association between quality of FP counseling received in the past 24 months and modern contraceptive use, initiation, and continuation among married women who were not already using any permanent method of contraceptive, and who did not report discontinuation because they wanted to become pregnant, in rural Uttar Pradesh, India. We measured quality of FP counseling using four items - whether the woman was informed of advantages and disadvantages of different methods, was told of method(s) appropriate for her, whether her questions were answered, and whether she perceived the counseling to be helpful. Positive responses to every item was categorized as higher quality counseling, vs lower quality counseling for positive responses to less than four items. The minority of participants had received any FP counseling (22%); only 4% received higher-quality counseling. Those who received any FP counseling (both lower- and higher-quality) had higher odds of reporting current use of any modern contraceptive, as compared to women reporting no FP counseling. Similar associations were observed with initiation and continuation of modern contraceptives. Findings suggest the value of quality FP counseling. With FP counseling, continued use of modern contraceptives can be promoted among married women of reproductive age.

## Background

Half of the currently married women aged 15–49 years in India use modern contraceptives, with 70% of all users choosing female sterilization [1]. The use of modern short-acting contraceptives (e.g. pill, condom) and Long Acting Reversible Contraceptives (LARC) (e.g. Intrauterine Device/Post-partum Intrauterine Device [IUDs/PPIUDs]) remain low (15.2%) [1]. Contraceptive discontinuation rates are also high for most available methods in India, with 25.5% of women who use modern non-permanent contraceptives reporting discontinuation for reasons other than pregnancy/fertility. This is a major concern, given that only 11.4% of married women and sexually active unmarried women of childbearing age in the country report any non-permanent contraceptive use [1]. Improving the use of modern contraceptives has potential to reduce unintended pregnancy and short inter-pregnancy intervals, which in turn can lead to reduction in adverse health consequences for mothers and infants [2, 3].

In response to this low prevalence of contraceptive use in India, recent efforts have tried to address barriers to access, with strategies to strengthen quality of family planning counseling provided [4]. Globally however, the discourse on quality of FP counseling was initiated nearly three decades ago, when Bruce [5] developed a theoretical framework on quality of FP services. The framework includes aspects of contraceptive technology safety, method mix options, technical competency, two-way exchange in provision of information, management of side effects, follow up care including method or provider switching, and integration with other reproductive health services. More recent work on quality of FP services emphasize interpersonal relations, or individual’s experiences, including aspects such as counseling being respectful and considerate of the couple and women’s dignity, privacy and confidentiality, as well as, practical consideration of readiness of service to provide the quality of care that is intended at the policy level [6, 7]. Providing high quality and respectful contraceptive counseling is important to support modern contraceptive use and meet couples’ family planning needs and goals [8–10]. Prior studies on specific contraceptive methods show that characteristics of FP counseling that are associated with use and continuation of contraceptive methods include proper counseling on side effects and information [11–16], clarification of misconceptions [17, 18], and addressing spousal dynamics like covert use and communication [19–21]. Studies also suggest the importance of counseling that provide opportunities for information exchange to support a choice that fits the reproductive needs and goals of the patient [6, 22, 23].

Despite the early and ongoing global work on quality of FP counseling services, few studies in low and middle-income countries (LMICs) such as India have tried to empirically assess quality of FP counseling [24–26] and its association with contraceptive initiation and continuation. The few studies that have examined this association have largely limited analysis to specific reversible methods of contraceptives rather than overall prevalence of modern spacing contraceptive use [24, 27]. To our knowledge, no study has looked at this issue longitudinally to better consider potential effects of quality FP counseling rather than just its association with better outcomes. The purpose of this study was to assess whether receipt of quality of FP counseling received in past 24 months is associated with subsequent modern contraceptive prevalence (mCP) with a longitudinal sample of married women in rural Uttar Pradesh, India. Secondarily, the study also examined whether quality FP counseling was associated with subsequent initiation and continuation of any modern contraceptive among a longitudinal sample of women in rural India. The study site, Uttar Pradesh (UP) is the most populous state in India, with low non-permanent modern contraceptive use of 24.7% [1]. This study has timely policy implications, given the recent expansion of FP services in India to prioritize LARC (IUDs/PPIUDs and injectables) and increased investments to prioritize FP in UP [28, 29].

## Methods

We used data from a longitudinal household survey conducted across 49 districts of UP, India. We used a multistage sampling design to obtain a representative sample of currently married women in the reproductive age (15–49 years), also described elsewhere [30]. A census of all the households from each selected primary sampling unit was conducted to identify the respondents who were married women of reproductive age (15–49 years). During the first round of the survey conducted from June–September 2014, 2400 women were approached for an interview, and 2222 of them consented (92.6% participation rate). Using contact information collected at baseline, the women still residing in the selected areas were interviewed during the second round of the longitudinal study, conducted from August–October 2016. Of the eligible longitudinal respondents, 1801 (80% completion rate) consented.

Since the analysis focused on initiation and continuation of modern contraceptives during the two rounds of survey, we excluded four groups of women from the analysis: women who were more than 49 years of age at the second round of survey (*n* = 31 women), women who were already sterilized at baseline (*n* = 329 women), women whose husbands were already sterilized at baseline (*n* = 13 women), and women who reported discontinuation of contraceptive use because they wanted to become pregnant (*n* = 30 women). We decided to exclude women who reported a desire to become pregnant, in order to examine the effects of FP counseling among women who have a potential need for contraceptives. We thus had a total sample of 1398 women for analysis. We weighted the data based on the sampling design to produce estimates representative of the selected geographies.

Trained female staff obtained formal informed consent and then collected data from participants using mobile tablets. These staff members were trained on accurate and sensitive collection of demographic and reproductive health related information. There was no contraceptive counseling, or contraceptive provision interventions delivered as study activities. Study protocols were reviewed and approved by Public Health Service-Ethical Review Board (an independent ethical review board) and the Health Ministry Screening Committee Indian Council for Medical Research.

### Measures

The survey tools used in this study were based on formative research and expert input, and were pilot tested prior to implementation. For the first analysis examining the relationship between FP counseling and modern contraceptive use, our main outcome of interest was *mCP*, which assessed use of any modern method of contraceptive reported at the second round of the survey. For the second analysis assessing the relationship between FP counseling, and initiation and continuation of modern contraceptive, our main outcome assessed method use behavior during the two rounds of survey. We categorized women as: a) *Continued users-* women who were using any modern contraceptive method during both the survey rounds b) *New users-* women who were not using any modern method during the first round but were using one during the second round of survey, c) *Discontinued users-* women who discontinued using any modern method from first to second round and d) *Non-users-* women who were not using any modern contraceptive method in either survey round. Modern contraceptive methods used by the eligible sample of women included Oral Contraceptive Pill, IUD (including PPIUD), injectable, implants, male and female condoms, lactational amenorrhea method, diaphragm and emergency contraceptive pill.

The key independent variable was *quality of FP counseling,* which assessed any experience with FP counseling during the 2 years between the two rounds of survey. The variable was categorized as: a) *No counseling, b) Receipt of lower quality counseling* and *c) Receipt of higher quality counseling*. *No counseling* was defined by answering no to the question, ‘In the last 2 years, did you receive any counseling / advice on FP from any community health worker, or health provider at a health facility?’ Among those who reported to have received any counseling based on the above question, the following four yes/no questions were used to capture quality of the counseling received:

1. Were you given information about the FP methods available, including the advantages and disadvantages of the methods?
2. Did the health service provider exchange information with you about which FP method(s) would be most appropriate for you?
3. Would you say the health service provider was able to address all your/your husband’s questions?
4. Would you say the counseling helped you in choosing the right method for you?

A positive response to all four items was categorized as *higher quality counseling,* vs *lower quality counseling* for a positive response to less than four items*.* Existing literature guided the development of the measure for quality of counseling, consistent with the framework of rights-based approach to counseling by Bruce [5, 6]. FP and survey measure experts within the research team constructed the questions and vetted them with FP experts external to the team. These efforts were designed to ensure face validity of these items. The measure was tested for internal reliability (Cronbach alpha = 0.87). The women who reported discontinuing using any modern contraceptive method were also asked about the reasons for discontinuation.

The covariates for the regression models were selected based on their conceptual importance, indicated by evidence from previous studies in India [31, 32]. We then examined the independent association of each covariate with the outcome, as well as the key independent variable. To adopt a parsimonious model, the variables with established relevance, and with *p* value less than 0.25 for unadjusted models were considered as covariates [33]. Final covariates included in the models were grouped as a) reproductive history related variables, and b) socio-demographics. Reproductive history measures included age at first childbirth (continuous variable), birth parity (continuous variable) and whether the respondent had any living sons. The variable on presence of any living sons was added to account for India’s known history of son preference [34]. Socio-demographic measures included age at marriage (continuous variable), age of woman (continuous variable), caste, religion, household wealth (continuous variable), women’s literacy status and husband’s education. Caste was categorized as Scheduled Caste/Scheduled Tribe (SC/ST), Other Backward Class (OBC) and General. The variable on caste was added as numerous studies indicate caste-based health inequities in India; those belonging to SC/ST and OBC have adverse mortality as well as morbidity outcomes as compared to those belonging to General or OC [35]. Religion was dichotomized as Muslim and Hindu/Others. The Standard of Living Index (SLI) was used as a proxy indicator for characterizing household wealth; the SLI methodology has been used for this purpose in the Demographic and Health Surveys across multiple national contexts, including India [36]. The SLI scores were calculated by summing the weights of 27 items indicative of an individual’s living standards, including consumer durables, housing conditions and access to basic services such as water and electricity. SLI scores were then normalized. A woman was considered literate if she reported being able to both read and write in at least one language. The proxy used for husband’s education was whether he had completed primary education, i.e. completed school till 5th grade.

### Statistical analysis

We used descriptive analysis to examine the continuation and discontinuation pattern for specific contraceptive methods between the two rounds of survey. We used bivariate and multivariate logistic regression models to assess the association between FP counseling experience and any modern contraceptive use during the second round of the survey. Next, we used multivariate multinomial logistic regression models to assess the effects of counseling (no counseling, lower quality, and higher quality counseling) on initiation, continuation or discontinuation of any modern contraceptive. Multinomial logistic regression models were used when the outcome variable has more than two categories [37]. We estimated adjusted relative risk ratio (RRR) from our multinomial models; RRR indicates the ratio between the exposure and reference category of the independent variable, of the risk of the outcome being categorized as the comparison group, as compared to the risk of the outcome being categorized as the referent group. For our analysis, we considered *No counselling* as the reference category for the independent variable, and *Non-users*, or women who were not using any modern contraceptive method in both the rounds of survey, as the reference outcome category.

Additionally, we ran a limited-sample multinomial logistic regression model for the sample of women who reported to have received any counseling. We used this model to examine the effect of higher quality counseling on contraceptive continuation and initiation, as compared to those that received lower quality counseling. The total sample for this model was 310 women.

We used sample weights calculated based on the multistage sampling design in all analyses. All data was analysed using STATA 15.0 software (StataCorp, USA) [38].

## Results

Table 1 shows the characteristics of women in the study (*N* = 1398). More than half (70%) were non-users of any modern contraceptive during the two rounds of survey. A similar percentage of women were continued users, new users and discontinued users (10, 11, and 10% respectively). Approximately, 22% of the women received any counseling on FP and only 4% received a higher quality counseling. With regards to the four items assessing quality, 17% of women were informed of the advantages and disadvantages of different methods, 9% exchanged information facilitating choice of an appropriate method, 12% reported that their questions were addressed by the provider, and 6% reported that the counseling helped them in choosing the method that was appropriate for them. A majority of the women who received some counseling during the 2 years between the surveys, received it from a community health worker (96%). Around 1% received it from any public or private health professional.

**Table 1:** Sample characteristics of married women of reproductive age in rural UP at the second round of survey (*N* = 1398)

Characteristics	% (95% CI) / Median (IQR)
Modern contraceptive use behavior	
Continued use	9.63 (7.40–12.44)
New use	10.86 (8.45–13.83)
Discontinued use	9.79 (7.34–12.95)
Consistent non-use	69.72 (65.83–73.35)
FP counseling experience (during 2 years between the surveys)	
No counseling	78.23 (74.15–81.83)
Lower quality counseling	18.09 (14.80–21.93)
Higher quality counseling	3.67 (2.58–5.20)
*Reproductive history*	
Age at first birth (continuous)	20.00 (19.00–23.00)
Birth parity (continuous)	3.00 (2.00–5.00)
Any living son	
No	19.20 (16.23–22.58)
Yes	80.80 (77.42–83.77)
*Socio-demographics*	
Age at marriage (continuous)	15.00(15.00–16.00)
Age of woman (continuous)	33.00 (27.00–40.00)
Caste	
SC/ST	23.36 (19.59–27.61)
OBC	59.49 (54.70–64.12)
General	17.15 (13.59–21.40)
Religion	
Hindu/Others	80.35 (74.63–85.03)
Muslim	19.65 (14.97–25.37)
Wealth index (continuous)	
Literacy	
Illiterate	66.25 (62.60–69.70)
Literate	33.75 (30.30–37.40)
Husband’s education	
Not completed primary education	33.99 (29.81–38.44)
Completed primary education	66.01 (61.56–70.19)

The median age of marriage was 15 years and the median age for giving birth to first child was 20 years. Around one-third of the sample were literate (34%).

Among the eligible women, around one-half were not using any modern or traditional contraceptive method during the first round of the survey (Table 2). Around 12% used condoms, 4% used pills and less than 1% used IUDs in the first round of the study. Five percent women were pregnant in round 1. The majority of women were also not using any method during round 2 of the survey. One-half of the condom users continued using condoms. Seven percent of condom users switched to oral contraceptive pills; a similar percentage of pill users switched to condoms. All except one woman continued using IUD during the second round of survey. Most LAM users either stopped using any method or switched to traditional methods. Among the round 1 non-users, most who initiated any contraceptive method used condoms, followed by pills.

**Table 2:** Percentage of married women of reproductive age using different contraceptive methods in round 2, by different methods used in round 1 (*N* = 1398)

	Round 2 unwtd. % of users (unwtd. n)								
		Female sterilization	Condoms	Pills	IUD	LAM	Other modern methods^a^	Traditional methods	No use
Round 1	Condoms (12.23%)	0	53.22 (91)	7.02 (12)	1.17 (40)	0	0	14.62 (25)	23.98 (41)
	Pills (4.22%)	0	6.78 (4)	42.37 (25)	0	0	0	13.56 (8)	37.29 (22)
	IUD (0.43%)	0	0	0	83.33 (6)	0	0	0	16.67 (1)
	LAM (3.00%)	0.05	2.38 (1)	14.28 (6)	0	0	0	35.71 (15)	42.86 (18)
	Other modern methods (0.65%)	0	11.11 (1)	0	0	0	22.22 (40)	0	66.67 (6)
	Traditional methods (23.82%)	0	10.51 (35)	3.90 (13)	1.20 (4)	0.30 (1)	0.30 (1)	45.95 (153)	37.84 (126)
	No use (Not pregnant) (50.43%)	0.57 (4)	6.24 (44)	4.11 (29)	0.99 (7)	0.57 (4)	0.55 (4)	21.28 (150)	65.67 (463)
	No use (Pregnant) (5.22%)	0	5.48 (4)	6.85 (5)	1.37 (1)	0	0	13.70 (10)	72.60 (53)

^a^Includes implants, injectables and female condoms

The most common reasons for discontinuation among those that reported discontinuation of modern methods, included, infrequent sex (57%), inconvenience (13%), difficulty in accessing contraceptives (12%), concerns regarding health effects/side effects of contraceptive use (12%), wanting a more effective method (11%), spousal disapproval (9%), and difficulty in using a method (1%).

Receiving any counseling during the 2 years after the first round of the survey was associated with an increased the odds of using any modern method of contraceptive during the second round of the survey (Table 3). Women receiving lower quality counseling had twice the odds of using any modern method (AOR: 2.42; 95% CI: 1.56–3.76) whereas women receiving higher quality counseling had four times the odds of using any method (AOR: 4.14; 95% CI: 1.72–9.99), as compared to women who did not receive any counseling, after adjusting for all covariates. Among socio-demographic characteristics, wealth index was associated with increased odds of using of any modern contraceptives. We also observed a trend towards association between religion and husband’s literacy and decreased use of modern contraceptives, but these associations did not meet statistical significance (AOR: 0.64; 95% CI: 0.38–1.07) and (AOR:0.72; 95% CI: 0.49–1.05) respectively.

**Table 3:** Bivariate and multivariate logistic regression to assess relationship between counseling and contraceptive use during round 2 of survey (*N* = 1398)

	Unadjusted OR (95% CI)	Adjusted OR (95% CI)
FP counseling experience (during 2 years between the surveys)		
No counselling	REF	REF
Lower quality counseling	2.11 (1.40–3.18) ***	2.42 (1.56–3.76) ***
Higher quality counseling	4.07 (1.79–9.26) ***	4.14 (1.72–9.99) ***
*Reproductive history*		
Age at first birth (continuous)	0.99 (0.98–1.004)	0.99 (0.98–1.00)
Birth parity (continuous)	0.92 (0.84–1.006) *	0.93 (0.82–1.03)
Any living son		
No	REF	REF
Yes	0.95 (0.57–1.55)	1.19 (0.62–1.83)
*Socio-demographics*		
Age at marriage (continuous)	1.04 (0.94–1.16)	1.02 (0.90–1.16)
Age of woman (continuous)	0.99 (0.96–1.01)	1.00 (0.97–1.02)
Caste		
SC/ST	REF	REF
OBC	0.99 (0.64–1.55)	0.99 (0.65–1.49)
General	1.56 (0.98–2.47) *	1.21 (0.75–1.95)
Religion		
Hindu/Others	REF	REF
Muslim	0.62 (0.37–1.01) *	0.64 (0.38–1.07) *
Wealth index (continuous)	1.01 (1.003–1.02) **	1.01 (1.001–1.02) **
Literacy		
Illiterate	REF	REF
Literate	1.46 (0.96–2.24) *	1.11 (0.71–1.72)
Husband’s education		
Not completed primary education	REF	REF
Completed primary education	1.13 (0.75–1.69)	0.72 (0.49–1.05) *

*p < 0.10 ** p < 0.05 ***p < 0.01

Table 4 presents results from the multinomial logistic regression models. We observed statistically significant association between receipt of FP counseling and contraceptive continuation, as well as initiation, when compared to consistent non-use of contraceptives. Higher quality counselling had higher adjusted relative risk ratio for continued contraceptive method use (ARRR: 5.93; 95% CI: 1.97–17.83), which implied that those receiving higher quality counseling were more likely, when compared to those that did not receive any counseling, to continue using a modern contraceptive against not using a modern contraceptive consistently for 2 years during the study period. We found similar association between receipt of higher quality counseling and being a new user, as compared to being a consistent non-user (ARRR: 4.22; 95% CI: 1.44–12.35). Those receiving lower quality counseling were also more likely, when compared to those that did not receive any counseling, to continue using a contraceptive (ARRR: 2.22; 95% CI: 1.08–4.52), and initiate the use of a method (ARRR: 2.94; 95% CI: 1.78–4.15).

**Table 4:** Multinomial logistic regression models to assess association between counseling and contraceptive use behavior [Reference category for multinomial regression: Consistent non-use of modern contraceptives] (N:1398)

	Continued users AOR (95% CI)	New users AOR (95% CI)	Discontinued users AOR (95% CI)
FP counseling experience (during 2 years between the surveys)			
No counseling	REF	REF	REF
Lower quality counseling	2.22 (1.08–4.52) **	2.94 (1.78–4.15) ***	1.35 (0.68–2.71)
Higher quality counseling	5.93 (1.97–17.83) ***	4.22 (1.44–12.35) ***	2.25 (0.61–8.20)
*Reproductive history*			
Age at first birth (continuous)	1.00(0.97–1.02)	1.00 (0.98–1.01)	1.01 (0.98–1.03)
Birth parity (continuous)	0.91 (0.78–1.06)	0.95 (0.82–1.08)	0.98 (0.84–1.14)
Any living son			
No	REF	REF	REF
Yes	2.05 (0.91–4.58) *	0.81 (0.39–1.66)	1.38 (0.60–3.18)
*Socio-demographics*			
Age at marriage (continuous)	1.05 (0.88–1.25)	0.97 (0.84–1.12)	0.93 (0.73–1.17)
Age of woman (continuous)	0.99 (0.96–1.02)	1.02 (0.98–1.05)	1.103 (0.98–1.07)
Caste			
SC/ST	REF	REF	REF
OBC	1.08 (0.52–2.26)	0.72 (0.44–1.18)	0.47 (0.25–0.87) **
General	1.78 (0.77–4.08)	1.03 (0.56–1.90)	0.90 (0.40–1.98)
Religion			
Hindu/Others	REF	REF	REF
Muslim	0.85 (0.41–1.74)	0.58 (0.29–1.19)	0.77 (0.38–1.54)
Wealth index (continuous)	1.01 (0.99–1.03)	1.01 (0.99–1.02)	1.00 (0.98–1.01)
Literacy			
Illiterate	REF	REF	REF
Literate	1.18 (0.66–2.08)	1.15 (0.66–1.97)	1.62 (0.94–2.78)
Husband’s education			
Not completed primary education	REF	REF	REF
Completed primary education	1.16 (0.62–2.15)	0.64 (0.38–1.08) *	1.34 (0.72–2.49)

*p < 0.10 ** p < 0.05 ***p < 0.01

With respect to other covariates, there were trends observed for presence of a living son and continued contraceptive use (ARRR 2.05; 95% CI: 0.91–4.58). Husband’s education was associated with lower likelihood of new use of modern contraceptives (ARRR:0.64; 95% CI: 0.38–1.08).

The limited-sample models comparing the receipt of higher quality counseling with lower quality counseling showed no statistically significant association between higher quality counseling and either of the positive outcome categories (Table 5). (ARRR: 2.65; 95% CI: 0.73–9.59 for continued use, and ARRR: 1.21; 95% CI: 0.35–4.09 for new use, as compared to consistent non-use).

**Table 5:** Multinomial logistic regression models to assess association between lower and higher counseling and contraceptive use behavior [Reference category for multinomial regression: Consistent non-use of modern contraceptives] (N:310)

	Continued users AOR (95% CI)	New users AOR (95% CI)	Discontinued users AOR (95% CI)
FP counseling experience (during 2 years between the surveys)			
Lower quality counseling	REF	REF	REF
Higher quality counseling	2.65 (0.73–9.59)	1.21 (0.35–4.09)	1.39 (0.32–6.09)

Note: Model adjusted for all covariates

## Discussion

We found significant associations between FP counseling, both lower and higher quality counseling, with mCP, as well as initiation and continuation of modern contraception. Approximately half of users of non-permanent modern contraceptive methods discontinued using them. Most of these women switched to either no use or traditional methods of contraception, which are less effective, and can increase the risk of unintended pregnancies. Our results are in line with, and build upon prior studies which find that challenges with access, inconvenience of use, and concerns about side effects and health effects are some of the key reasons for discontinuation of modern methods [39]. Taken together, our results suggest that FP counseling is important to help women achieve their reproductive goals, and to support method initiation and continuation. We found that higher quality counseling is not significantly different from lower quality counseling, with respect to their effect on contraceptive continuation and initiation. It is however important to note that the sample of women who received any counseling was low, with an even lower number of women receiving higher quality counseling. Our findings of trends between higher quality counseling and contraceptive use highlight the need for a greater focus on expanding quality FP services to more women.

Previous data suggests that treating women with respect, and utilizing shared decision-making in the counseling process can lead to improved contraceptive uptake and continuation, and return for care if dissatisfied with a method [6, 40, 41]. Our study adds to this literature by highlighting the value of quality counseling to support successful contraceptive continuation among women in a LMIC. Given that mistreatment and coercion of women by their healthcare providers is not uncommon [42, 43], and target-driven global FP goals for LMICs like India can yield coercive practices [44], assurance of quality FP counseling in the rapidly expanding availability of services in the country should be prioritized.

Multiple recent studies have focused on assessing quality of FP counseling empirically in LMICs [24, 25]. Holt et al. [45] validated a quality of contraceptive counseling scale to improve measurement of client experiences with providers in two Mexican states. Another study conducted in two states of India validated a process quality measure which captures the information exchange and interpersonal relations between a provider and a client, and tested their predictive validity related to continued use of reversible contraceptive methods [24]. Our study contributes to this rapidly evolving literature on measuring quality of FP counseling. By using a short measure, we examine the prevalence of any and higher quality FP counseling in rural India, and its effects on continuation and initiation of any modern method of contraceptive. The four questions used to develop our FP quality measure capture aspects of interpersonal experience of counseling - delivery of required information by the provider, and provision of a supportive environment for clarification and information exchange [6].

It is notable that most of the women receiving counseling received it from a Community Health Worker, also known as ASHAs [Accredited Social Health Activist] in India. This is reflective of the programmatic focus on task sharing for care in India, with the implementation of national-level interventions which have designated ASHAs to carry out doorstep FP counseling and delivery of contraceptives [46]. Findings demonstrate that ASHAs do increase access to FP counseling, but a large proportion of women are still excluded from these services. It indicates the importance of better support for ASHAs if they are to provide the majority of FP counseling. However, it is important to note that for uptake of methods such as IUDs, provision requires a clinically-trained health provider to initiate use. Our study demonstrates that a very small proportion of women (less than 2%) received any counseling from clinicians, suggesting a huge gap in provision of FP services.

A strength of our study is its longitudinal design, which allows for an exploration of method switching and assessment of temporal relationships. Among those not using any permanent methods, the most prevalent method of modern contraception used were condoms, followed by oral contraceptive pills. The discontinuation rates were, however, high for both the methods. This is consistent with prior studies in India which have indicated frequent discontinuation of these short-acting methods [47]. LARC methods like IUDs, on the other hand, have very low prevalence but discontinuation is much lower among these users. Studies have suggested numerous reasons for the low prevalence of LARC in India, which include difficulty in access or low availability, inadequate knowledge and fear and misconceptions regarding its side-effects [48, 49]. Low knowledge about IUDs gives rise to various misconceptions which has led to its low prevalence in India [48]. To this end, quality counseling might support an improvement in LARC uptake. It is important to ensure that low discontinuation is due to high acceptability rather than inadequate counseling about method discontinuation for IUDs [6]. Another key finding of our study is that most women using LAM as the method of contraception postpartum later switched to either no or traditional method of contraception. FP counseling in the postpartum period can be used as an effective time to support women to transition to another modern method of contraceptive and prevent short inter-pregnancy intervals. A recent study in India found provision of FP counseling to women during their stay at the health facility for childbirth to be associated with increased modern contraceptive use 1 year later [50].

Although this current study provides important insights into the effect of quality of FP counseling on contraceptive initiation and continuation, there are a few limitations. First, the study is susceptible to the presence of recall bias and social desirability bias due to use of interviewer-administered questionnaires and self-reported measures for contraceptive use and counseling. Alternative methods to measure quality of FP care such as exit interviews, counseling session observations, or using mystery clients couple help triangulate and substantiate findings from traditional measures [51]. Second, while we captured elements of quality of counseling, we were limited by availability of data on few aspects of quality of counseling, including, specifically asking if providers elicited client needs and preferences, need-concordant choice, and follow-up visits. Third, the study did not include any information on who initiated conversations around the specific items of counseling, the woman or the provider. Fourth, the study did not gather any data on timing and frequency of counseling, which might have an impact on contraceptive initiation. The effect of counseling on contraceptive use could be an overestimate due to greater interest among the women engaged with FP counsellors, limiting our understanding of contraceptive acceptability. Fourth, prior to asking the questions about receipt of counseling, the study did not enquire whether the woman initiated any contact or interaction with a reproductive health provider during the time period between the two surveys. Our study thus examines the effects of receipt of counseling, and does not comment on whether it was the woman who sought any services related to reproductive health. This is one key limitation of our study. However, for our study geography, i.e. India, prior research has shown that existing social norms often prevent women from seeking reproductive health services when they want to [52]. Additionally, current prevalence of receipt of FP counseling among married women is extremely low, as shown by our analysis. In this context, we believe it is important to examine the effect that receipt of counseling will have on contraceptive use behavior, at a population level, irrespective of whether it was the woman or health provider who initiated the interaction. Due to limitations of sample size, we could not carry out a method-specific analysis, FP counseling might have differential effects on use and continuation of different methods. Finally, the findings are applicable to women in India and are not generalizable to other geographies.

## Conclusions

Findings from the study highlight the value of FP counseling to support increased modern contraceptive use and reduce discontinuation in a context such as UP, India where contraceptive use remains low. With high quality and a person-centered approach to counseling, continued use of modern contraceptives can be supported among married women of reproductive age. Unfortunately, FP counseling, particularly higher quality FP counseling, remains rare in UP. These findings highlight the importance of access to, and delivery of, quality FP counseling via highly trained CHWs and clinical providers.

## Data Availability

The dataset supporting the conclusions of this article is available in the Harvard Dataverse, https://dataverse.harvard.edu/dataset.xhtml?persistentId=doi:10.7910/DVN/GBOAIG.

## Abbreviations

ASHA: Accredited Social Health Activist
FP: Family Planning
IUD: Intrauterine Device
LAM: Lactational amenorrhea method
LARC: Long Acting Reversible Contraceptives
mCP: Modern Contraceptive Prevalence
PPIUD: Post-partum Intrauterine Device
UP: Uttar Pradesh
